# Microbial Pre-exposure and Vectorial Competence of *Anopheles* Mosquitoes

**DOI:** 10.3389/fcimb.2017.00508

**Published:** 2017-12-07

**Authors:** Constentin Dieme, Brice Rotureau, Christian Mitri

**Affiliations:** ^1^Genetics and Genomics of Insect Vectors Unit, Department of Parasites and Insect Vectors, Institut Pasteur, Paris, France; ^2^Centre National de la Recherche Scientifique Unit of Hosts, Vectors and Pathogens (URA3012), Paris, France; ^3^Trypanosome Transmission Group, Trypanosome Cell Biology Unit, Institut National de la Santé et de la Recherche Médicale U1201 and Department of Parasites and Insect Vectors, Institut Pasteur, Paris, France

**Keywords:** *Anopheles*, *Plasmodium*, malaria, vectorial competence, microbial pre-exposure

## Abstract

*Anopheles* female mosquitoes can transmit *Plasmodium*, the malaria parasite. During their aquatic life, wild *Anopheles* mosquito larvae are exposed to a huge diversity of microbes present in their breeding sites. Later, adult females often take successive blood meals that might also carry different micro-organisms, including parasites, bacteria, and viruses. Therefore, prior to *Plasmodium* ingestion, the mosquito biology could be modulated at different life stages by a suite of microbes present in larval breeding sites, as well as in the adult environment. In this article, we highlight several naturally relevant scenarios of *Anopheles* microbial pre-exposure that we assume might impact mosquito vectorial competence for the malaria parasite: (i) larval microbial exposures; (ii) protist co-infections; (iii) virus co-infections; and (iv) pathogenic bacteria co-infections. In addition, significant behavioral changes in African *Anopheles* vectors have been associated with increasing insecticide resistance. We discuss how these ethological modifications may also increase the repertoire of microbes to which mosquitoes could be exposed, and that might also influence their vectorial competence. Studying *Plasmodium–Anopheles* interactions in natural microbial environments would efficiently contribute to refining the transmission risks.

## Introduction

Vector-borne diseases are a major cause of human mortality and morbidity in the World, among which malaria is a prominent threat. The two-thirds of the global population at risk for malaria infection are found in sub Saharan Africa, where 1.5–2.5 million deaths are reported every year (Vernick, 2004; Mitri et al., 2009; McGraw and O'Neill, 2013; Silbermayr et al., 2013; Smith et al., 2014; Blank et al., 2015). The devastating human malaria parasite, *Plasmodium falciparum*, is strictly transmitted by insect vectors of the genus *Anopheles* that are a necessary link in the chain of human-to-human malaria transmission (Vernick, 2004; Smith et al., 2014). *Anopheles* and *Plasmodium* genetics play a major role in determining mosquito vector competence (Riehle et al., 2006; Mitri et al., 2009; Harris et al., 2010). Other determinants such as the mosquito immunity, the ambient temperature, the mosquito diet and its microbial gut flora also play important roles in mosquito–parasite interactions (Molina-Cruz et al., 2012; Lefevre et al., 2013; Medeiros et al., 2014).

Permanent and temporary bodies of water that serve as larval habitat for wild mosquitoes are rich in diverse microbes. These larval site microbes could interact with the immune system of mosquito larvae in multiple ways and likely influence the pattern of immune activation in the future emerging adults (Dimopoulos et al., 1997; Gimonneau et al., 2012). Furthermore, adult female mosquitoes require successive blood meals for egg production and these bloodmeals may also contain not only *Plasmodium* parasites, but other micro-organisms (e.g., other parasites, viruses, bacteria) (Scott and Takken, 2012). Hence, prior to *Plasmodium* ingestion, the mosquito biology could be modulated at different life stages, from the egg to the young adult, by a wide range of microbes present in larval breeding sites, as well as in the adult ecosystem. Indeed, in areas where mammalian hosts carry diverse microbes, any adult mosquito female could theoretically be exposed to a wide range of organisms prior to, or together with, the ingestion of *Plasmodium*.

In the literature, effects of co-infections and exposure to multiple microbes have been intensely investigated in mammalian hosts, however, such studies remain scarce in their arthropod hosts (Dennison et al., 2014; Zélé et al., 2014a,b). Here, we first propose several relevant scenarios highlighting microbial exposure of *Anopheles* mosquito vectors that might impact their vectorial competence for *Plasmodium*. Then, considering that increasing insecticide resistance has been associated with significant changes in mosquito behavior (Gatton et al., 2013), we discuss how these ethological modifications might also impact the suite of microbes to which mosquitoes could be exposed, and that might also influence their vectorial competence. Overall, this opinion article aims at highlighting the benefit of a better understanding of the *Plasmodium*–*Anopheles* interaction systems in a more natural scheme that better consider relevant microbial ecosystems, a step that would be crucial for refining the transmission risk mapping and designing novel vector control strategies.

## Larval microbial exposure

Mosquito oviposition and larval developmental stages are aquatic for about 10–15 days depending on the species, the temperature and the feeding resources. In nature, the breeding sites are rich in co-habiting microbes that could infect mosquito larvae and we reasoned that these dynamic and virtually limitless microbial repertoires might activate the immune system of the larvae and probably of the emerging adults too. A recent study provides evidences that *Aedes* larval exposure to different bacteria can drive variation in adult traits underlying vectorial competence for dengue virus (Dickson et al., 2017). In Cameroun, larval habits were dominated by Acinetobacteria (12–16%), Firmicutes (11–24%), and Protobacteria (54–74%) phylum (Gimonneau et al., 2014). Interestingly exposition of *Anopheles gambiae* with natural midgut bacteria such as *Escherichia coli, Serratia marcescens*, and *Pseudomonas stutzeri*, which all belong to the most abundant Proteobacteria phylum, negatively impact on *Plasmodium* infection (Tchioffo et al., 2013).

**Figure 1:**
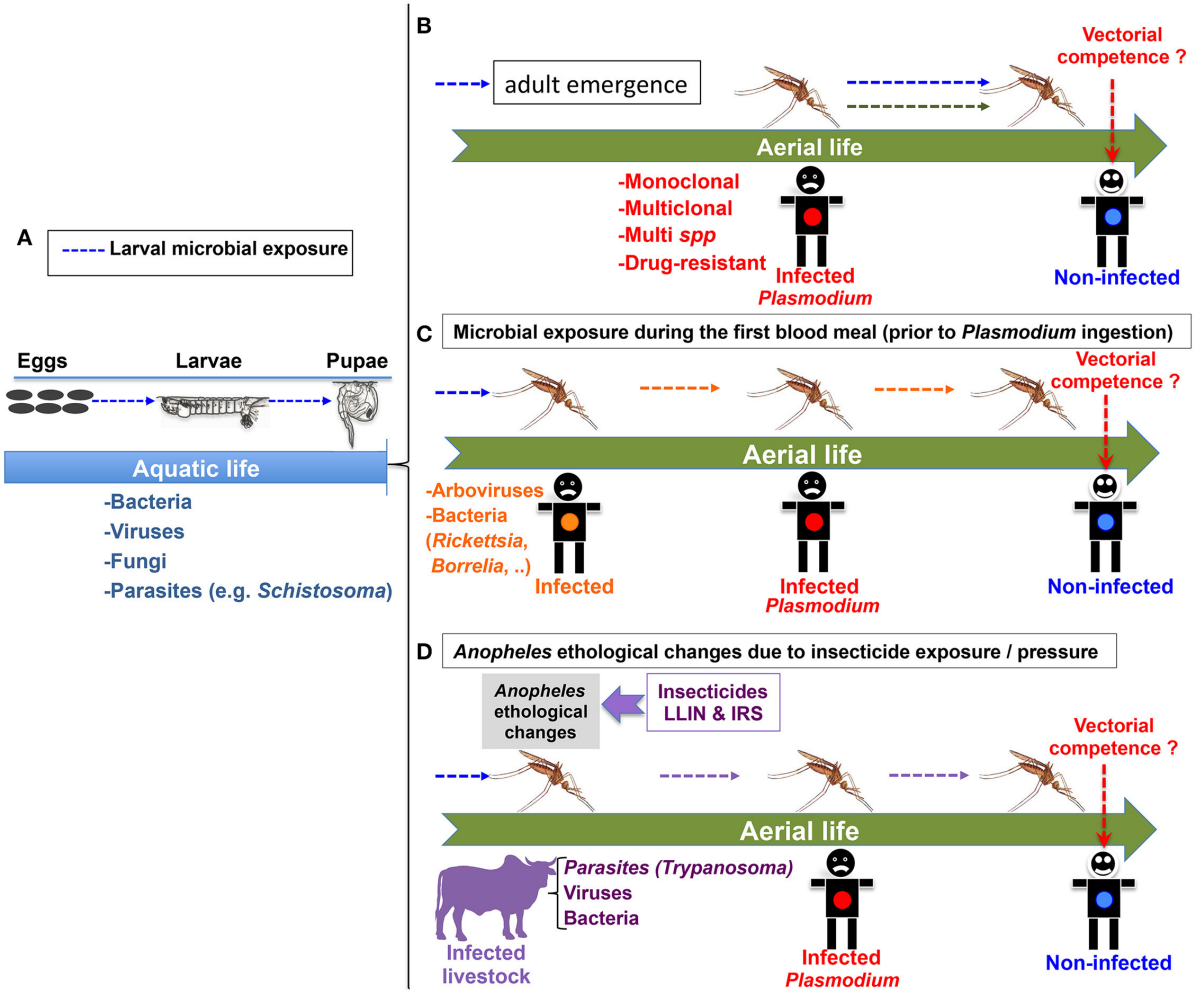
Different scenarios for microbial pre-exposure of *Anopheles* mosquitoes that might impact their vectorial competence for the malaria parasite *Plasmodium*. **(A)** Larval microbial exposure (blue arrows) could impact the vectorial competence of future adult mosquito females. **(B)** Microbial exposure during the larval stages could impact the vectorial competence of adult mosquito females (blue arrows). The green arrow represents the possible effect of a multi-clonal or multi-species (including drug resistant strains) *Plasmodium* ingestion on the vectorial competence of *Anopheles*. **(C)** Microbial exposure (in orange) during the first blood meal could modulate vectorial competence. The human reservoir with an orange dot could be infected with diverse microbes. **(D)** One example of environmental change: *Anopheles* ethological changes resulting from insecticide exposure/pressure may increase the repertoire of microbial pre-exposure, as *Anopheles* mosquitoes could adapt their feeding preferences and feed on infected animals possibly carrying a wide range of microbes. For **(B–D):** The cartooned man with red dot is infected with *Plasmodium* gametocytes. The one with a blue dot is not infected, but could potentially become infected depending on the vectorial competence of the co-infected vector.

The characterization of these microbial communities in the mosquito larval habitat remains scarce in the literature. However with the recent development of next generation sequencing (NGS) technologies, such studies will become more affordable for identifying new bacteria (Gimonneau et al., 2014), viruses, as well as protists (Belda et al., 2017) communities that might specifically shape the immune system of the mosquito larvae and probably the vectorial competence of the emerging *Anopheles* adults (Figures 1A,B).

## Protist co-infections in *Anopheles* mosquitoes

### Multiple *Plasmodium* clones and species

Several studies have shown that humans co-infected with distinct clones of *P. falciparum* are common in regions of high malaria transmission (Smith et al., 1999; Joshi et al., 2007). This also means that *Anopheles* mosquitoes biting a human host that carries multiple clones of *Plasmodium* gametocytes will have to deal simultaneously with parasites of distinct genetic backgrounds. It was reported that monoclonal infections of *P. falciparum* are efficiently controlled by the mosquito immune system whereas multi-clonal infections tend to escape from the mosquito immune surveillance (Nsango et al., 2012). How distinct *Plasmodium* clones and/or species cohabit and/or compete with each other in different *Anopheles* genetic background, and what the consequences of such co-habitations are on the parasite transmission remain important questions that have been poorly addressed. One noticeable exception study has identified a new axis of mosquito resistance to monoclonal *P. falciparum* infections that includes the AP-1 transcription factor Fos and the transglutaminase 2 (TGase2) (Nsango et al., 2013).

In addition, very few studies have investigated the impact of *Plasmodium*-drug resistance on *Anopheles* vectorial competence. Artemisinin-based combination therapies are now the World Health Organization (WHO) recommended first-line treatment for *P. falciparum* malaria in all countries with endemic disease. However, there are increasing concerns about artemisinin-resistant strains of *P. falciparum* in Asia (Kone et al., 2010; Amaratunga et al., 2012; Roper et al., 2014), and more recently in Africa (Roper et al., 2014; Lu et al., 2017; Madamet et al., 2017). Furthermore, detection of *Plasmodium* gametocytes in artemisinin-resistant strains indicates that they can be transmitted to the mosquito vector (Tun et al., 2015). In a *Plasmodium*-multi-clonal scenario, *Anopheles* mosquitoes could ingest both sensitive and resistant clones simultaneously or successively. Therefore, it will be important to know the impact of drug-resistant parasites on transmission of drug sensitive *Plasmodium* by *Anopheles* mosquitoes.

Human malaria was known to be caused by five *Plasmodium* species: *P. falciparum, Plasmodium vivax, Plasmodium malariae, Plasmodium ovale* and since 2008, *Plasmodium knowlesi* has been considered as the fifth human malaria parasite (Cox-Singh and Singh, 2008). A longitudinal study performed in a holoendemic area of Senegal showed that in all seasons, *An. gambiae s.l* and *Anopheles funestus* are often simultaneously infected by two or three species of *Plasmodium* (Trape et al., 1994). Although the most deadly human malaria parasite, *P. falciparum*, is highly studied, *P. vivax* also has attracted a broad interest because it is widely distributed and causes recurring malaria. It was initially thought that a high proportion of African people are resistant to *P. vivax* because they lack the Duffy antigen required for *P. vivax* erythrocyte invasion. However, recent studies have indicated that Duffy-negative people were actually not fully protected against *P. vivax* (Ryan et al., 2006; Woldearegai et al., 2013; Lover, 2014; Gunalan et al., 2016). Indeed, clinical *vivax* malaria was identified in Duffy-negative subjects with *P. vivax* mono-infections, but also in co-infections with other *Plasmodium* species (Menard et al., 2010). In Kenya, *An. gambiae* and *An. funestus* were found positive for the *P. vivax* circumsporozoite protein, suggesting potential transmission of *P. vivax* by African malaria vectors (Ryan et al., 2006). Therefore, co-infection by multi-*Plasmodium* species in mosquitoes could occur more frequently than previously thought in Africa. How these distinct *Plasmodium* species interact within *Anopheles*, and what this means for vectorial competence for each parasite species is unknown (Figure 1B). To tackle this issue, one could, for instance, feed *Anopheles* mosquitoes on blood from patient carrying both *P. falciparum* and *P. vivax*, and further monitor the sporogonic development of each parasite species in the vector using a qPCR approach to detect species-specific gene target.

### Plasmodium and trypanosoma

Sleeping sickness (Human African Trypanosomiasis) and nagana (Animal African Trypanosomiases) are neglected tropical diseases caused by flagellated protists of the *Trypanosoma* genus that are transmitted by a specific blood feeding insect vectors, the tsetse fly. Nagana has devastating socio-economic consequences on animal production in Africa (Rotureau and Van Den Abbeele, 2013). African trypanosomes circulate in 37 sub-Saharan countries where approximately 70 million people and about 50 million cattle are currently living at risk of infection. Less than 3,000 new human cases were reported to WHO in 2015, 98% of which being caused by *Trypanosoma brucei gambiense* (Buscher et al., 2017). In many areas, tsetse flies and mosquitoes currently share the same habitat, and both human and animal African trypanosomiases occur in areas where malaria is also endemic. Blum et al. reported a malaria prevalence of 50% in *T.b. gambiense* patients from several endemic countries (Blum et al., 2006). In Alupe Kenya, between 2000 and 2009 amongst 31 HAT cases recorded from the National Sleeping Sickness Referral Hospital, 100% were found co-infected with malaria parasites (Kagira et al., 2011). Given that trophic behavioral changes have been recently discovered in *Anopheles* mosquitoes initially described as exclusively anthropophilic (see section below), the occurrence of successive infections by *Trypanosoma* and *Plasmodium* in *Anopheles* mosquitoes may increase to measureable frequencies. It is therefore important to carry out studies to determine the impact of such co-infections on the natural cyclical development of *Plasmodium* in *Anopheles* mosquitoes, and consequently on its transmission (Figure 1D).

### Plasmodium and helminths

Schistosomiasis kills 20,000 to 200,000 people every year (WHO_Fact_Sheet_N°115, 2016)^1^ The disease is most commonly found in Africa, as well as in Asia and South America, and is due to a human parasite called *Schistosoma* (WHO_Fact_Sheet_N°115, 2016)^1^. *Schistosoma* larvae develop in an aquatic environment. Parasite eggs are laid in water within human feces or urine. After hatching, eggs release miracidia (~150 μm), the free-swimming larval stages that infect a specific snail (the intermediate host), in which the parasites develop into sporocysts and finally into cercariae. Swimming cercariae released from snails are the only infective forms for humans. Malaria and schistosomiasis are co-endemic in many areas of Africa, and co-infections can occur in humans (Brooker et al., 2007; Naing et al., 2013). A study performed in Ethiopia showed that among malaria febrile patients, 38% were co-infected with *S. mansoni* (Degarege et al., 2012). Moreover, coinfections with *Plasmodium* and *Schistosoma* infection have known implications for the health of children of all ages in sub-Saharan Africa (Sokhna et al., 2004; Wilson and Dunne, 2012). It is noteworthy that the aquatic environment is a common phase for the development of both schistosome and mosquito larvae. Although it is poorly documented, *Schistosoma* host snails and mosquito larvae are commonly found in the same larval sites (Ofulla et al., 2010). Therefore, assuming that mosquito larvae are exposed to *Schistosoma* miracidia in co-endemic areas, it could be interesting to assess how this exposure could affect the mosquito biology, especially their survival at the larval stages (i.e., the rate of successful adult emergence) as well as their susceptibility to *Plasmodium* in adult mosquitoes previously exposed to miracidia during their larval stage (Figure 1A). The miracidia (150 μm) could be ingested by *Anopheles* larvae or attached to the larval cuticle. In the latter case, we could not exclude the possibility for the miracidia to penetrate the cuticle of mosquito larvae: using a genetically modified parasite expressing a fluorescent protein could help assessing this issue. For the ingestion scenario, it is known that excretory-secretory products released during transformation of miracidia to sporocyst in the invertebrate host snail, can impair phagocytosis and reduce the production of reactive oxygen species from snail's haemocytes (Connors and Yoshino, 1990). Therefore assuming that miracidia could reach the larvae midgut, excretory-secretory (ES) products might also affect the mosquito physiology e.g., by modifying the midgut environment and/or the larval bacteria flora. Other possible effects of these ES products, as described in the host snail (Lockyer et al., 2012; Pinaud et al., 2016), could be long-term modifications of the larvae immune system that might also affect the vector competence of the emerging adult.

Others helminthiasis, such as lymphatic filariasis (LF) and onchocerciasis due to *Wuchereria bancrofti* and *Onchocerca volvulus* respectively are also co-endemic with malaria (Brooker et al., 2007; Muturi et al., 2008). Although *O. volvulus* is transmitted to the human host by the black fly and potentially by *Aedes* (Zielke et al., 1977), *Anopheles* mosquito are known to be competent vector for the parasite responsible for LF (Boakye et al., 2004; Muturi et al., 2008). Therefore, *W. bancrofti* could theoretically be in contact with *Plasmodium* parasites ingested with another blood meal. In this particular case, future studies could tackle how the successive or concomitant presence of the two parasites (*Wuchereria* and *Plasmodium*) could affect their respective transmission by *Anopheles* mosquito vectors.

## *Plasmodium* and viruses co-infections in *Anopheles* mosquitoes

### *Plasmodium* and arboviruses

Although arboviruses, such as dengue, chikungunya, or yellow fever viruses, and *Plasmodium* infections differ in their development cycles and transmission dynamics, some similar ecological traits suggest that interactions between these micro-organisms could occur within their hosts. Several reports described arbovirus and *Plasmodium* co-infections in humans throughout the World (Epelboin et al., 2012; Medeiros et al., 2014), e.g., malaria and dengue mixed infections are common in the field (Carme et al., 2009). Their co-circulation within a given human host population increases the probability of concurrent mosquito vector infections (Medeiros et al., 2014). Mosquitoes can become co-infected when they bite a human host harboring both malaria parasites and arboviruses. Another scenario by which mosquitoes may become infected by both *Plasmodium* spp. and arboviruses is consecutive bites on two different singly infected hosts. In addition, field caught malaria vectors such as *An. funestus* and *Anopheles coustani* have been found to carry arboviruses such as Chikungunya, Zika, and yellow fever viruses, emphasizing the plausibility of *Anopheles* exposition to arboviruses (Sow et al., 2016). *Anopheles coluzzi* is also the vector of the O'nyong nyong arbovirus, against which it develops an immune response that is accompanied by peculiar modifications and activities of the bacterial gut flora (Carissimo et al., 2015). While *Anopheles* mosquitoes are not important vectors for the majority of arboviruses, the sole fact of being exposed to these viruses, even for periods as short as 12–24 h, could significantly impact on their biology, especially in the midgut. For instance, Dengue virus serotype-2 (DENV-2) can be detected in *An. coluzzi* until 24 h post ingestion (Unpublished result). *Anopheles* midgut is known to be the first organ where *Plasmodium* starts its sporogonic development, and is also the first bottleneck for the parasite population in the mosquito (Han et al., 2000). Any arbovirus could potentially immune-prime *Anopheles* mosquito and further impact on the invasion and development of *Plasmodium* (Medeiros et al., 2014) (Figure 1C). For example, one could perform a first infectious feeding of *Anopheles* females with DENV, followed with a second infectious blood meal containing *Plasmodium* parasites 3–4 days later. In the control group, the first blood meal should be non-infected. This experiment would allow assessing whether DENV pre-exposure could impact the vectorial competence of *Anopheles* for *Plasmodium*.

### Plasmodium and HIV

The human immunodeficiency virus (HIV) is a lentivirus that causes HIV infection and over time acquired immunodeficiency syndrome (AIDS) (Weiss, 1993). HIV continues to be a major global public health issue, having claimed more than 35 million lives so far. In 2016, 1.0 million people died from HIV-related causes globally (WHO_Fact_Sheet_HIV/AIDS, 2017)^2^ The African region, which is the most affected with 25.6 million people living with HIV, accounts for almost two thirds of the new HIV infections (WHO_Fact_Sheet_HIV/AIDS, 2017)^2^ HIV-1 and *P. falciparum* malaria therefore remain two of the major causes of morbidity and mortality in Sub-Saharan Africa where they are sympatric (Andreani et al., 2012; Orlov et al., 2012). In a population of about 200,000 people that have been exposed to both pathogens since 1980 in Kenya, the interaction of the two diseases may have caused 8,500 excess HIV infections and 980,000 excess malaria episodes (Abu-Raddad et al., 2006). Many studies have focused on the impact of the simultaneous presence of these two pathogens (HIV and *Plasmodium*) on their respective development in humans, and on the severity of the diseases (AIDS and malaria), however the effect of the presence of both pathogens in *Anopheles* mosquitoes is not known. While the HIV virus is not transmitted by mosquitoes, it is noteworthy that it has a short lifespan in their digestive tract (48 h) (Bockarie and Paru, 1996; Iqbal, 1999). Therefore, during this time frame (48 h), proteins from the virion surface could induce an immune response in *Anopheles* mosquitoes biting on a co-infected human host, which may further impact on the development of *Plasmodium*. Chemical inactivation of the HIV-1 virus has been shown to be immunogenic (Rossio et al., 1998), suggesting that the conformation of the surface proteins from inactivated virions is still intact. To further assess the potential impact of HIV surface proteins on the sporogonic development of *Plasmodium* in *Anopheles*, chemically inactivated HIV-1 could be mixed with *Plasmodium* gametocytes to co-infect *Anopheles* mosquitoes.

## Malaria and bacteria co-infections in mosquitoes

The mosquito midgut can possibly contain some pathogens harmful to humans, but also a complex ecosystem composed by diverse bacterial communities that may vary depending on the geographical origin, the ecological niche, the season and the source of food (Dennison et al., 2014; Tchioffo et al., 2015). Mosquitoes are continuously exposed to a variety of microbes originating from both their aquatic and aerial ecosystems; some of these microbes develop a symbiotic relationship with the insect, while others are commensal or pathogenic (Dennison et al., 2014).

### Domesticated bacteria gut

There is strong evidence that bacteria influence mosquito susceptibility to human pathogens as well as their capacity to transmit them (Dong et al., 2009; Boissiere et al., 2012; Saraiva et al., 2016). Recent studies have suggested that microbiota might influence *Plasmodium* development directly through the production of antimalarial compounds, or more indirectly by maintaining basal immune activity, or through resources competition (Dennison et al., 2014; Smith et al., 2014; Saraiva et al., 2016). Interestingly, co-feeding of an Enterobacter bacterium isolated from wild mosquito populations in Zambia, with *Plasmodium* gametocytes, renders *Anopheles* mosquitoes resistant to human malaria *P. falciparum* parasites through the production of reactive oxygen species (Cirimotich et al., 2011).

*Wolbachia*, is a Gram-negative endosymbiont commonly found in Arthropods (in 60% of insects). Although present in several mosquito species, including *Culex pipiens* and *Aedes Albopictus, Wolbachia* is not naturally present in any anopheline species that transmitsmalaria parasites, nor in *Ae. aegypti* (vector of the dengue virus). *Wolbachia* from *Drosophila* fruit flies, strain *w*MelPop, or from the mosquito *Ae. Albopictus*, strain *w*Alb, have experimentally been introduced in *Ae. aegypti* and *Anopheles* species. Each of these *Wolbachia* strains, introduced in *Ae. aegypti*, reduces the ability of certain viruses, such as dengue and chikungunya viruses, to develop in these mosquitoes (Moreira et al., 2009; Walker et al., 2011; Mousson et al., 2012). However, the effects of *Wolbachia* on *Plasmodium* development in the mosquito are not fully understood (Hughes et al., 2014). Introduction of both *Wolbachia strains wMelPop* and *wAlb* in *An. stephensi* mosquitoes induced their refractoriness to the human malaria parasite *P. falciparum* (Bian et al., 2013). However, the opposite effect is observed when *An. gambiae* mosquitoes carrying the *w*AlB strain are infected with the rodent malaria parasite *Plasmodium berghei* (Hughes et al., 2012). Similarly, another study showed that the *Wolbachia wPip(Sl)* strain increases the susceptibility of *C. pipiens* mosquitoes to the avian parasite *Plasmodium relictum* (Zélé et al., 2014a), suggesting that *Wolbachia*-infected mosquitoes may also have an opposite effect to that observed on the vectorial competence of *Aedes* and *Anopheles* for dengue and *P. falciparum*, respectively. The reasons for such contrasting results remain unknown. One potential explanation is the disparity of the immune modulation mediated by different strains of *Wolbacchia* that could, in turn, differentially impact the pathogenic microbes transmitted by different species of mosquito vectors. Another reason could be the impact of *Wolbachia* on the midgut environment, and probably on the entire composition of the microbiota in the mosquito midgut (McGraw and O'Neill, 2013; Smith et al., 2014; Zélé et al., 2014a,b). A recent study indicated that *Wolbachia*-altered cholesterol is responsible for the viral refractoriness in *Aedes* cells (Geoghegan et al., 2017). The *Plasmodium* parasite is unable to synthesize cholesterol *de novo*, as it can access cholesterol from the host via either the endogenous or exogenous pathways (Labaied et al., 2011). Therefore, we cannot exclude that, like in *Aedes* cells, *Wolbachia* may alter the lipid metabolism of *Anopheles* mosquitoes, which in turn may inhibit the sporogonic development of *Plasmodium* in the mosquito host. Moreover, a novel *Wolbachia* strain, distinct from strains infecting other arthropods, has been identified in *Anopheles gambiae* from Burkina Faso, West Africa (Baldini et al., 2014). This emphasizes the need for further investigations on the possible use of natural *Wolbachia*-*Anopheles* associations to limit malaria transmission.

In Cairns, Australia, *Wolbachia* has been successfully spread through mosquito populations, in order to reduce the transmission of dengue and zika viruses (Jiggins, 2017). However, due to the inconsistent effects of *Wolbachia* on the transmission of distinct pathogens, such strategies for controlling vector competence should be cautiously implemented and monitored in the field (Figure 1C).

### Pathogenic bacteria

A large panel of tick-borne diseases, which affect both humans and wild mammals, can result from the transmission of several pathogens including bacteria, viruses, and protists. It is known that ticks can harbor and co-transmit multiple pathogens (Knapp and Rice, 2015). Malaria and tick-borne diseases overlap in many African regions, therefore co-infections in *Anopheles* mosquitoes may be possible after successive or concomitant infectious blood meals on hosts carrying both micro-organisms. In African malaria-endemic regions, bacteria such as *Rickettsia, Borrelia, Bartonella*, and *Coxiella* are often the cause of misdiagnosed malaria-like fever cases (Mediannikov et al., 2013; Fotso Fotso and Drancourt, 2015; Mourembou et al., 2015; Angelakis et al., 2016; Cutler et al., 2016; Sothmann et al., 2017). In Sub-Saharan Africa, *Borrelia-*induced mortality rate is estimated at between 2 and 5% (Fotso Fotso and Drancourt, 2015), and in rural Senegal, the incidence of tick-borne relapsing fever is estimated to reach 11% (Fotso Fotso and Drancourt, 2015; Cutler et al., 2016). Rickettsioses are a major public health problem in sub-Saharan Africa (Mediannikov et al., 2013; Mourembou et al., 2015; Sothmann et al., 2017). *Rickettsia felis* and *Plasmodium* co-infections were found in humans (Mediannikov et al., 2013) and recent studies showed that mosquitoes can readily transmit pathogenic bacteria such as *R. felis* and *Francisella tularensis* (Backman et al., 2015; Dieme et al., 2015). Therefore, *Anopheles* mosquito co-infections with *P. falciparum* and *Rickettsia, Borrelia, Bartonella*, and/or *Coxiella* may occur in significant proportions in sub-Saharan Africa. How this pre-exposure or co-infection status affects the vectorial competence of *Anopheles* for *Plasmodium* is not known and certainly requires future work (Figures 1A,C). Bacteria such as *Rickettsia* and *Borrelia* can easily be cultivated and quantified (Bonnet et al., 2015; Dieme et al., 2015), and could therefore be mixed within blood for performing an experimental infection of *Anopheles* female. These females would then take a *Plasmodium*-infected blood meal to evaluate the impact of bacterial pre-exposure on their vectorial competence for *Plasmodium* as compared to non-exposed females.

## Environmental changes and *Anopheles* microbial exposure

### Effects of insecticide exposures on *Anopheles* trophic behavior

Since 2000, prevention of malaria in Africa is largely based on vector control and especially on the individual use of insecticides in the domestic environment with either long-lasting insecticide-treated bednets (LLINs) or indoor residual spraying (IRS) (Bhatt et al., 2015; Hemingway et al., 2016; Ranson and Lissenden, 2016). These measures have significantly reduced the mortality and morbidity of malaria in Africa by 40% in 15 years (Bhatt et al., 2015). However, resistance against pyrethroids (the only chemical class used for LLINs) are widely documented in *An. gambiae* and *An. funestus* in sub-Saharan Africa (Kawada et al., 2011; Silva et al., 2014). In addition, increased resistance to pyrethroids, carbamates, and organophosphates that are routinely used in IRS programs are also observed (Aizoun et al., 2013; Riveron et al., 2016; Ondeto et al., 2017). This decrease in insecticide efficiency led to an increase of the vector population size (Bhatt et al., 2015; Hemingway et al., 2016; Ranson and Lissenden, 2016) and some studies have reported the presence of sporozoites in mosquitoes containing insecticides resistant alleles, which may significantly compromises the elimination of malaria (Alout et al., 2013; Kabula et al., 2016).

With the increasing indoor use of both LLIN and IRS, *Anopheles* mosquitoes tend to adapt by finding other sources of blood, especially outside housings. There are strong evidence that *Anopheles* behavior is shifting from indoor to outdoor biting, or from night to dawn biting in areas where LLINs are used (Russell et al., 2011; McGraw and O'Neill, 2013; Ranson and Lissenden, 2016). This strongly suggests that insecticide exposure has probably influenced mosquito behavior and fitness. *An. gambiae*, which is the World most important vector for *Plasmodium* parasites, was initially described to show a high degree of anthropophily, coupled with strong endophilic and endophagic traits (Scott and Takken, 2012; Takken and Verhulst, 2013). However, recent studies on its feeding behavior have shown high proportions (i) of blood meals actually taken on a single non-human host (animal) and (ii) of mixed blood meals taken from both animals and humans (Diatta et al., 1998; Ngom et al., 2013). These behavioral changes may enlarge the diversity of the microbial repertoire to which *Anopheles* mosquitoes are exposed, and concomitantly increase the probability of co-infections with *Plasmodium*. Therefore, we suggest that the increasing risk of microbial pre-exposure related to these ethological changes might also impact on the vectorial competence of *Anopheles* for the malaria parasite (Figure 1D).

### Effect of climate changes on *Anopheles* biology

Several studies have been conducted to evaluate the impact of temperature variation and climate change on the vectorial competence of *Anopheles* for *Plasmodium* (Paaijmans et al., 2010; Gething et al., 2011; Caminade et al., 2014; Murdock et al., 2016). A recent study has investigated how temperature could affect several mosquito-parasite traits such as mortality rate, parasite extrinsic incubation period, and biting rate (Shapiro et al., 2017). While all these studies raise important issues related to the effect of temperature on *Anopheles* vectorial competence, the impact on *Anopheles* microbial exposure was not assessed.

Nevertheless, the biting rate evaluated in (Shapiro et al., 2017) could be extrapolated to microbial exposures, assuming that each biting event exposes the mosquito to microbes. In that sense, we could therefore consider that temperature variation may indirectly affect *Anopheles* microbial exposure. In addition, the temperature of a mosquito body is very closed to ambient temperature, hence it would also be interesting to assess whether external temperature variation might affect the composition of the bacterial gut flora, which is known to influence *Anopheles* vectorial competence. Similarly, larval ecological niches subjected to temperature variation might also be impacted for their microbial compositions. This could modulate the immune system of the larvae and possibly influence the pattern of immune activation in the emerging adults, and overall their vectorial competence. Further studies are needed to tackle all these different issues.

### Effect of mosquito diet

Sources of sugar for mosquitoes include floral nectar, extra-floral nectaries, and plant fluids (Impoinvil et al., 2004; Muller et al., 2010; Gouagna et al., 2014; Faiman et al., 2017). Dietary-restricted organisms across many taxa have been reported to survive longer than those with unlimited or normal access to food (Vellai et al., 2003; Hatle et al., 2006; Bjedov et al., 2010). A recent study has investigated the effect of dietary restriction on *A. coluzzi* lifespan and showed that sugar restriction significantly enhances longevity of the mosquitoes (Faiman et al., 2017). Extension of female longevity might also increase the number of blood feeding and therefore the probability of microbial exposure events.

Furthermore, several drugs such as antibiotics (Gendrin et al., 2015) or Ivermectine (Kobylinski et al., 2012), present in ingested mammal blood, were shown to affect *Anopheles* vector competence for *Plasmodium*. Therefore it would also be interesting to consider these aspects for studying the *Anopheles-Plasmodium* system in its natural environment.

## Conclusion

*Anopheles* mosquitoes are continuously exposed to a wide range of microbes from the larval aquatic stages to the aerial adult stage. In the latest stage, the obligate hematophagy of the female mosquitoes that need to take successive blood meal on multiple hosts enlarge the repertoire of microbes to which they are exposed, including *Plasmodium*, the deadliest parasite in the World. Many studies focusing on the *Anopheles*–*Plasmodium* interaction have highlighted several mosquito factors controlling the susceptibility of the *Anopheles* mosquito female to *Plasmodium*. However, these studies have been more often performed in controlled biological systems that considered the mosquito as infected only with the *Plasmodium* parasite. Microbial exposure of *Anopheles* mosquito, prior to, or concomitant with *Plasmodium* ingestion, could significantly modify the vectorial competence of *Anopheles* for *Plasmodium* in distinct ways, e.g., via resource competition, modification of the midgut environment and of the bacterial gut flora, immune priming, releasing factors that might interfere directly or indirectly with the *Plasmodium* parasites. Figure 1 summarizes several relevant scenarios of microbial pre-exposure that could significantly impact anopheline biology, and consequently modulate their vectorial competence for *Plasmodium*. We assume that studying the *Anopheles-Plasmodium* system in its natural and dynamic environment, including relevant microbial pre-exposures, would refine our understanding of the complex mechanisms ruling the vector competence. This would be more relevant and certainly helpful for elaborating optimal antivectorial strategies.

## Author contributions

CD, BR, and CM designed the plan, wrote, and discussed the manuscript.

### Conflict of interest statement

The authors declare that the research was conducted in the absence of any commercial or financial relationships that could be construed as a potential conflict of interest. The reviewer AM and handling Editor declared their shared affiliation.

## Footnotes

^1^http://www.who.int/mediacentre/factsheets/fs115/en/

^2^http://www.who.int/mediacentre/factsheets/fs360/en/
